# Tabes Dorsalis and Syphilis

**Published:** 1905-07-22

**Authors:** 


### TABES DORSALIS AND SYPHILIS.

In a recent lecture Sir Wm. Gowers[1] sums up the evidence bearing on the alleged relationship between tabes dorsalis and syphilis. The proposition is that the organisms of syphilis produce a toxine analagous to the nerve poison operative in diphtheritic paralysis, and that this toxine is responsible for the morbid process and symptoms of tabes. The morbid process, however, is not a true specific lesion but a simple nerve degeneration, and hence tabes is not a direct but an indirect result of syphilis. For these statements it cannot be said there is conclusive proof. But there is a strong presumption resting mainly on the sequence of observed facts. In a series of 1,100 cases recorded by Erb, and carefully investigated in reference to a possible syphilitic causation, a history of a primary sore was obtained in 89·5 per cent. Gowers himself found in several hundred private cases a history of a chancre in 80 per cent.; 15 per cent. denied a sore but had suffered from gonorrhœa, and of the remaining 5 per cent., everyone admitted having run risks of infection in the ordinary way. Hospital statistics give less suggestive testimony, but in the lower classes the facts are often forgotten, and further, the history of the cases in this direction is not always thoroughly explored. Eighty per cent. may at first sight appear to be too small a proportion on which to affirm a constant sequence. But there is reason to believe that syphilis may be communicated with urethritis, and thus some of the cases in which the latter disease was admitted may be included amongst the examples of specific affection. Certainly undoubted constitutional syphilis is sometimes seen in patients who deny all forms of venereal disease other than a gonorrhœa. An instructive series of facts illustrating the frequent absence of a history of primary disease in constitutional syphilis is found in some figures collected at University College Hospital by Mr. G. Pernet. These show that in 124 cases of skin disease, the syphilitic nature of which was beyond question, a history of the primary sore could only be obtained in 100—that is, in 80 per cent. The figures are thus practically parallel to those afforded by tabes dorsalis. Gowers further remarks that, excluding members of the medical profession, he has never seen a case of true tabes in a man who has not run risks of infection in the usual way, and that of no other disease of the nervous system can the same assertion be made. Evidence advanced against the conclusion that in cases of tabes dorsalis syphilis is an invariable antecedent may be put on one side. It is vitiated by the inclusion in the cases observed of females as well as males, and by a readiness to regard every immediate antecedent of the symptoms as sufficient to make any other antecedent negligible. Further evidence of the influence of syphilis in the causation of tabes is found in cases of "juvenile tabes." This is doubtless a rare affection, but it may have all the characteristic facts, including optic atrophy, observed in the adult, and in every instance there is or has been evidence of inherited syphilis. The occasional occurrence of marital tabes, both husband and wife suffering from the disease, and the husband having had syphilis before marriage, is another conspicuous illustration of the causal sequence and relationship. It may be that some other agent of unknown nature cooperates with that which syphilis produces in the actual determination of the onset and evolution of tabes dorsalis, and that in this way the prejudicial influence of the specific toxine is increased. But the facts compel the admission that it is to the poison of syphilis that the primary cause of tabes dorsalis must be referred.

[1] Brit. Med. Jour., July 8, 1905.

